# Effect of Anolyte on *S.* Typhimurium and *L. monocytogenes* Growth in Minced Pork and Beef Cuts

**DOI:** 10.3390/foods11030415

**Published:** 2022-01-31

**Authors:** Reda Riešutė, Joana Šalomskienė, Alvija Šalaševičienė, Irena Mačionienė

**Affiliations:** Food Institute, Kaunas University of Technology, Radvilėnų Avenue 19, LT-50292 Kaunas, Lithuania; joana.salomskiene@ktu.lt (J.Š.); alvija.salaseviciene@ktu.lt (A.Š.); irena.macioniene@ktu.lt (I.M.)

**Keywords:** anolyte, *Listeria monocytogenes*, *Salmonella* Typhimurium, meat cuts, minced pork

## Abstract

In this paper, anolyte is considered as a possible disinfectant for inhibiting the growth of bacteria in meat (beef cuts and minced pork). Meat cuts were contaminated with two concentrations of *L. monocytogenes* and *S.* Typhimurium, as these are the most common meat pathogens that are closely regulated by the EU, and treated with two different concentrations of anolyte: 20% for beef cuts and 18% for minced pork. Then, the total viable count (TVC), *L. monocytogenes* count and *S.* Typhimurium count were determined. In meat cuts and minced pork, anolyte was able to reduce TVC, *S.* Typhimurium and *L. monocytogenes* counts effectively, significantly decreasing *L. monocytogenes* and *S.* Typhimurium counts after spraying and throughout 29 days of incubation at 0–4 °C. TVC was reduced after spraying and for 10 days of incubation but later increased to be the same as before spraying with anolyte. Anolyte was effective when spraying beef cuts with a 20% solution for 60 s against pathogenic bacteria *L. monocytogenes* and *Salmonella* spp. and also when using it at a concentration of 18% from the minced meat mass. Initially, anolyte significantly decreased TVC, however during the storage period (10–29 days) TVC increased but remained significantly lower compared to control. Anolyte was effective in reducing *L. monocytogenes* and *S.* Typhimurium counts throughout the study, and after 29 days of incubation, these bacteria could not be detected in the samples treated with anolyte.

## 1. Introduction

Bacteria and yeasts play an important role in food spoilage. Thus, over the recent years, a lot of attention has been given to the control and prevention of their growth in food products to prolong their shelf life. The most commonly used means for preservation in the food industry are chlorinated water, organic acids, hydrogen peroxide, ozone and ultraviolet light. The problem with some of these materials is that they produce by-products that are toxic, so there is an emerging goal of finding a non-toxic, non-destructive method of disinfection [1,2].

In recent years, increased attention has been drawn to electrolyzed oxidizing water and its antimicrobial effects. Electrolyzed water (also called anolyte, acid water and non-living water) is gaining popularity as a sanitizer in the food industry of many countries. Anolyte is a colorless clear liquid with an acid scent. The anolyte dipping treatment was found to be as effective as chlorinated solutions in controlling the growth of aerobic bacteria, molds, yeasts and coliform bacteria during storage. Since the anolyte has the non-destructive properties of other organic materials, it is not harmful and can be used in food sanitation. Anolyte is effective and harmless, not only for the disinfection of food contact surfaces but also for the treatment (washing and spraying) of the products (vegetables, fruits, seafood and meat products) [3,4,5,6].

The production of anolyte has been outlined by Ignatov et al. [7]. Anolyte is produced through the process of electrolysis, where the cathode and the anode sides of the cell are separated by a diaphragm. This separates electrochemically activated water into alkaline, also known as catholyte, and acidic anolyte fractions. Catholyte is a powerful reducer, with ORP values less than −820 mV and a pH > 9, whereas anolyte is an oxidizer with ORP values over +800 mV and a pH < 7.3. ECA also possesses active components, such as Cl_2_, HCl, H+, OH− and others, obtained through the process of electrolysis.

The bactericidal action of a neutral anolyte is based on the oxidation of the substances of a bacterial cell, especially lipoprotein membranes, which are the only place of biosynthesis [8].

Although the mechanism for cell destruction is not yet fully understood, it is hypothesized that high ORP values over +810 mV create an unfavorable environment for bacteria to thrive in, causing links to break in cell structures. The change in membrane permeability allows the diffusion of oxidants to enter the cytoplasm, leading to the oxidation of proteins, which further leads to dysfunction and ultimately to the death of bacteria. The bactericidal effect, although greatly reduced, carries over to spores as well [9,10]. These findings were in line with research conducted by Kiura et al. [4] who found that electrolyzed strong acid water (ESW) created breaks and blebs in the cell membranes of *Pseudomonas aeruginosa*. These breaks, as well as the destruction of DNA, increased with growing concentrations of free chlorine concentrations. The conclusion was made that ESW is inclined to destroy the cell walls of Gram-negative bacteria. This was further confirmed by Rajkowski and Sommers [11], who noticed that Gram-negative bacteria seem to have a more sensitive reaction to anolyte, as shown by their research.

It has been found that undiluted anolyte effectively destroys *P. aeruginosa*, *Escherichia coli* and *Bacillus subtilis* immediately upon exposure. Diluted anolyte (10^−1^ dilution) produced similar results. However, *B. subtilis* was killed off after 6 h of exposure. Protein band analysis suggested cell protein destruction as the mechanism of action for killing bacteria [12]. The 5% anolyte concentration solution is a good disinfectant for mesophilic bacteria, yeasts and molds. Furthermore, it reduces the growth of bacteria after treatment [3]. Although anolyte was effective in reducing *Salmonella* cultures, there has been no significant difference in recovered populations of *L. monocytogenes* before and after treatment with anolyte. Although anolyte is shown to be harmful to bacterial cells, it does not seem to cause any health issues or cell destruction in humans [7]. Moreover, anolyte is environmentally safe, as it eventually returns to its original saltwater state [10]. Anolyte does not affect taste, smell or the properties of organic matter. Therefore, it is a perfect sanitizer for usage in the food industry. A study using the anolyte dipping method for the disinfection of dates [3] found that anolyte did not affect sensory descriptors or the biochemical contents of the fruit. Anolyte treatment also preserves the qualities of trout [13] as well as fruit [14]. Thorn et al. [10] also revealed a virucidal effect, as backed by other research papers, though those studies are argued to be insufficient for confirming anolyte use for viral infections [7].

Because *Salmonella* and *L. monocytogenes* are two of the main pathogens in food spoilage and anolyte could potentially be used for reducing bacteria counts, thus extending the shelf-life of meat, the aim of this study is to evaluate the effect of anolyte on the criteria of food safety for *Salmonella* and *L. monocytogenes* in semi-finished meat products, as these two bacteria have been identified as the main contaminants of meat and their levels in food products are tightly regulated in the EU [15].

## 2. Materials and Methods

The study was carried out at the microbiology research laboratory of the Food Institute of Kaunas University of Technology.

### 2.1. Bacterial Cultures

Two reference cultures of pathogenic bacteria, *Salmonella enterica* subsp. *enterica* serovar Typhimurium ATCC 14028 and *Listeria monocytogenes* ATCC 13932, were obtained from the American Type Culture Collection and used for contamination of model meat samples. Bacterial cultures were stored at the microbiology laboratory of the Food Institute of Kaunas University of Technology at minus 72–74 °C in a VIABANK system. Revitalization was performed in brain heart infusion broth (Liofilchem, Roseto, Italy).

### 2.2. Preparation of Bacterial Cultures for Contamination

*S. enterica* subsp. *enterica* serovar Typhimurium ATCC 14028 and *L. monocytogenes* ATCC 13932 cultures for the contamination of meat were grown on agar slants (Triptone Soya Agar. Liofilchem, Roseto, Italy) for 18 h at 37 °C. The target cultures were washed with sterile physiological solution and the density of cell suspension was adjusted according to the McFarland standard Nr. 0.5 (1.5 × 10^8^ cells/mL). Cell suspensions 1.5 × 10^3^ CFU/mL, 1.5 × 10^4^ CFU/mL and 1.5 × 10^5^ CFU/mL for meat contamination were prepared by diluting with sterile physiological solution. 

### 2.3. Preparation of Anolyte

Anolyte solutions were prepared by diluting anolyte with water that satisfied the requirements of drinking water. For minced pork, undiluted anolyte with the parameters of pH 6.56 and 182 ppm was used. 

### 2.4. Beef Cut Contamination and Anolyte Treatment

Good quality fresh beef was received from a meat processing plant (Lithuania).

Beef pieces were cut across the muscle into cuts of approximately 300 g by weight and 18–20 cm × 10–12 cm × 3 cm in size. The edge samples were not used for experiments. Untreated samples were put in a vacuumed package for 10 and 29 days (Control 1). 

For control 2 samples, ten milliliters of a bacteria suspension (*L. monocytogenes* ATCC 13932 or *S. enterica* subsp. *enterica* serovar Typhimurium ATCC 14028) were smeared onto the surface of the meat cut for its contamination. After 30–40 min, samples were taken and then the cuts were incubated in a vacuumed package for 29 days.

The unvacuumed beef cuts were hung up and sprayed continuously for 60 s with anolyte using a 0.5 L spray bottle, creating very fine drops of anolyte solution. After spraying with anolyte, the beef cuts were dried for 40 min at 5 ± 3 °C. Then, after taking samples, they were vacuum sealed for testing after 10 and 29 days of incubation at 0–4 °C. 

### 2.5. Minced Pork Contamination and Anolyte Treatment

Pork mince was received from a meat processing plant in Lithuania. After sampling controls, 200 g portions of minced meat were contaminated with *S. enterica* subsp. *enterica* serovar Typhimurium ATCC 14028 or *L. monocytogenes* ATCC 7644 suspension (10 mL of suspension of different concentrations), mixed with a Bosch MUM5 1000 W food mixer (BSH Home Appliances AB Vilnius, Vilnius, Lithuania). After taking samples, approximately 18% (36 mL) of the undiluted anolyte was added to the minced meat and mixed again. In 1 h, samples were taken again, and the minced meat samples were vacuum sealed for testing after 10 and 29 days of incubation at 0–4 °C.

### 2.6. Microbiological Analysis

The following microbiological analyses were performed:

- Total viable count (TVC) on plate count agar (Liofilchem, Italy);
- Number of *Salmonella* spp. on XLD agar, with colony confirmation using biochemical tests (reaction on triple sugar/iron agar, urea agar, L-lizine decarboxylation medium) and serological reaction if the colonies were suspicious or typical;
- Number of *Listeria* in Agar Listeria, according to Ottaviani and Agosti (Biolife, Monza, Italy), with colony confirmation using a microscopic view, beta-hemolysis test and L-Rhamnose and D-Xylose tests if the colonies were suspicious or typical.

### 2.7. Statistical Analysis

For each experiment, three trials were performed. The standard deviation was calculated using EXCEL (version 11. Microsoft, Washington, DC, USA) software. Data is given as an average value ± standard deviation. Differences between the data were evaluated by the analysis of variance method (one-way ANOVA) with a significance level of *p* < 0.05.

## 3. Results

No *L. monocytogenes* or *Salmonella* spp. were detected in any of the pieces of beef and pork obtained for testing.

Data on the investigation of 20% anolyte’s effect on total viable count (TVC) and pathogenic bacteria (*L. monocytogenes* and *Salmonella* spp.) count using the treatment of meat cuts by spraying are presented in Table 1, Table 2, Table 3 and Table 4. At 20% anolyte concentration, using the lowest contamination level (1500 CFU/mL suspension of *L. monocytogenes* and *S.* Typhimurium), two controls were used: Control 1 was raw meat not additionally contaminated by pathogenic bacteria and not treated by anolyte, and control 2 was raw meat contaminated by the analogous bacterial suspension used for experimental meat but not treated by anolyte. The experimental meat (for 20% anolyte treatment, beef) was contaminated by the uniform bacterial suspension (15,000 CFU/mL). 

According to the data in the Table 1, after spraying meat cuts treated at a 1500 CFU/mL contamination level with 20% anolyte, the TVC was reduced significantly (*p* < 0.05) from (4.73 ± 0.26) × 10^3^ to (4.30 ± 0.27) × 10^2^. However, it significantly increased after 10 days of incubation and increased even more after 29 days of incubation. Compared to Control 1, however, the TVC after 29 days was significantly lower in experimental meat, even with the increase in TVC compared to when meat was tested right after spraying with anolyte. The same could be seen at the higher contamination level (15,000 CFU/mL), where TVC was significantly reduced (12-fold decrease, *p* < 0.05) after spraying with anolyte, then increased after incubation. Despite the increase in TVC in experimental meat, the final TVC after 29 days was 1.3 times lower compared to the Control 1 and Control 2.

When using a lower contamination level (1500 CFU/mL), *L. monocytogenes* was reduced effectively to non-detectable levels, and these levels remained, even after 29 days of incubation. At the higher contamination level (15,000 CFU/mL), a similar situation could be seen, where the *L. monocytogenes* count was effectively kept at non-detectable levels after incubation. The anolyte took some time to fully reduce *L. monocytogenes,* as right after spraying with anolyte, the counts were reduced significantly. However, they still were at detectable levels ((3.33 ± 0.58) × 10^2^).

The results of Table 3 are analogous with ones presented in Table 1. Though treatment with anolyte decreased TVC on the meat surface significantly. The effect was not long lasting; after the incubation of samples for 10 and 29 days, the TVC increased up to (4.03 ± 0.06) × 10^6^ for the lower contamination level and (4.7 ± 0.1) × 10^6^ for the higher contamination level. As in Table 1, the final TVC after 29 days was lower in the experimental meat samples compared to control for both contamination levels.

The results of Table 4 are analogous with Table 2. All anolyte concentrations were effective in decreasing the *S.* Typhimurium count in the experimental samples, and it decreased during the incubation at 0–4 °C. *S.* Typhimurium were not detected after 29 days incubation.

The results of anolyte’s effect on the contamination of minced pork are presented in Table 5, Table 6, Table 7 and Table 8.

According to the data in Table 5, at different levels of contamination with *L. monocytogenes* (1.5 × 10^3^ CFU/mL, 1.5 × 10^4^ CFU/mL and 1.5 × 10^5^ CFU/ mL) of minced meat and treating with anolyte, the TVC decreased 2.2; 1.7 and 2.4-fold, respectively (*p* < 0.05). With the continued storage of meat samples at 0–4 °C for up to 29 days, the TVC significantly increased again by 35.0, 29.3 and 29.6 times. The final TVC was significantly lower (*p* < 0.05) in the lowest contamination sample and highest (*p* < 0.05) in the highest contamination sample.

According to the data in the Table 6, the *L. monocytogenes* count in the minced meat samples contaminated with these bacteria at different levels (1.5 × 10^4^ CFU/mL and 1.5 × 10^5^ CFU/mL) decreased by 6.2 and 19.2 times (*p* < 0.05), respectively, after exposure to 18% anolyte from the meat mass. The largest decrease was observed at the highest dose in the contaminated sample. However, the final TVC was still significantly higher in the highest contamination sample, whereas in the other two samples, *L. monocytogenes* was undetectable after 29 days.

According to the data in the Table 7, the TVC in samples 1, 2 and 3 (minced meat from the lowest to the highest contamination dose of *S*. Typhimurium) decreased by 3.4, 2.8 and 1.5 times, respectively (*p* < 0.05). When the samples were incubated at 0–4 °C for up to 10 days, the TVC increased significantly (*p* < 0.05), and after 29 days the TVC increased again by 64.1, 21.0 and 21.7 times (maximum increase at the lowest dose of contamination).

According to the data in the Table 8, in samples of minced meat contaminated with *S.* Typhimurium, *Salmonella* spp. counts decreased by 2.3 and 13.6 (*p* < 0.05) times in samples 2 and 3, respectively, after exposure to 18% pure anolyte from the meat mass and were not detected in sample 1 (at the lowest dose of contamination). When the samples were incubated at 0–4 °C for up to 29 days, the *Salmonella* spp. count in samples 2 and 3 decreased further by 16.3 and 6.1 times, respectively, and in the sample contaminated with the lowest dose they were not detected after 10 and 29 days of incubation. 

The TVC decreased upon exposure to anolyte in the minced meat by several times but increased again to maximum of 35-fold at the end of incubation (when contaminated *with L. monocytogenes*) and to 64-fold (when contaminated with *S*. Typhimurium). *L. monocytogenes* and *Salmonella* spp. count decreased after exposure to anolyte (*L. monocytogenes* maximum decreased 19-fold and *Salmonella* spp. count maximum decreased 13-fold) and was decreasing until the end of incubation and was not detected after contamination by lower concentrations.

## 4. Discussion

Rajkowski and Sommers [11] noticed similar results in their research, wherein catfish fillets inoculated with *Salmonella* and *L. monocytogenes* treated with anolyte retained a slight reduction in background microflora counts for only a 2 day storage period, yet *Salmonella* recovery from catfish fillet surface was reduced significantly and maintained throughout 13 days of storage. *L. monocytogenes* recovery was not reduced after a 3 min wash with anolyte. This is in accordance with our own findings, as *S.* Typhimurium counts were effectively reduced in beef cuts and minced pork and remained undetectable up to 29 days of storage at all contamination levels. Similar to their research, the TVC was initially reduced both in beef cuts and minced pork. However, it started increasing again through the storage period in our own research. In contrast to Rajkowski and Sommers’ paper, *L. monocytogenes* counts were also effectively reduced and kept at low levels during our research process. Similar studies have also been carried out with anolyte and its effect on *L. monocytogenes* in fish [16,17,18].

Fabrizio and Cutter [19] applied acidic electrolyzed water on ready-to-eat meats in an attempt to reduce the *L. monocytogenes* counts. The data of their research shows a positive effect of electrolyzed water in reducing *L. monocytogenes* counts in frankfurters following dipping treatments for up to 7 days, after which the bacteria counts started increasing again, yet still maintaining a lower count of bacteria compared to control. In contrast, in our research, *L. monocytogenes* counts kept reducing during the storage period up to 29 days, and at the end of the study were at undetectable levels at most contamination levels. The difference in results could be attributed to different exposure times between our research and Fabrizio and Cutter’s research; while in their research frankfurters were dipped in electrolyzed water for 15 min, anolyte in our study was mixed into the minced pork, leading to a longer exposure time for anolyte and, thus, leading to a better reduction in *L. monocytogenes* counts. Furthermore, acidic electrolyzed water was shown to be effective in reducing mesophilic, psychotrophic and lactic acid bacteria, which also contribute to meat spoilage [20].

Our results are also in concordance with Al-Holy and Rasco [17], who found that *S.* Typhimurium and *L. monocytogenes* populations in beef were reduced after a 10 min-long treatment with acidic electrolyzed water by 0.7 logs and 1.2 logs, respectively. The authors also found that AEW also reduced *E. coli* O157:H7 counts, which are also prominent bacteria in meat spoilage that were not studied in our paper. Liao et al. [21] determined that using slightly acidic electrolyzed water could also effectively control TVC levels in beef during thawing, as it damages cell structures and inhibits the growth of microbes on the surface of beef. 

Levels of meat contamination with *L. monocytogenes* and *Salmonella* Typhimurium even higher than those that occur in practice have been studied. In the latter case, the number of bacteria decreased under the impact of anolyte, and the data that was acquired in this study confirmed those described in the literature [22] that anolyte had a higher effect on pathogenic bacteria than on the TVC. In their paper, Hricova et al. [23] reviewed the efficacy of electrolyzed water in removing pathogenic bacteria. They note that anolyte is efficient in reducing *S.* Typhimurium and *L. monocytogenes* populations by more than 6.0 log CFU/mL and up to 9.2 log CFU/mL, respectively. Park et al. [24] reports a >4.0 log CFU/g reduction in pathogenic bacteria when using electrolyzed water compared to control, which reinforces the Navarro-Rico et al. [25] description of electrolyzed water as a “promising alternative to conventional NaClO disinfection”. In fact, the effect of anolyte depends on the species composition of the microorganisms present in meat. 

From the research, the following conclusions can be made:

- Anolyte is effective at reducing *L. monocytogenes* and *S.* Typhimurium counts in beef cuts and minced pork over a 29 day period;
- While initially anolyte can reduce the TVC in beef cuts and minced pork, it starts to increase during the storage period again. However, the TVC is lower, compared to control;
- Taking into account our findings and the findings of other authors, anolyte seems to be a promising tool for reducing bacterial growth in meat products and extending their shelf-life;
- In the future, research could be expanded further by studying the effects of anolyte on other pathogens, as well as in different types of meat and under different processing conditions.

## Figures and Tables

**Table 1:** The effect of anolyte’s concentration on the total viable count of meat cuts contaminated with *L. monocytogenes*.

Concentration of Anolyte Used for Spraying of Meat Cuts	Suspension of *L. monocytogenes* Used for Contamination of Meat Cuts 300 g, CFU/mL	Object of Investigation (Raw Meat)	Total Viable Count. CFU/g Meat Cut				
			Raw Meat	Meat after Contamination	Meat after Spraying with Anolyte	After 10 Days of Incubationat 0–4 °C	After 29 Days of Incubation at 0–4 °C
20%, beef	1500 CFU/mL	Control 1	(2.27 ± 0.06) × 10^3^	-	-	(1.97 ± 0.06) × 10^5^	(5.67 ± 0.29) × 10^6^
		Experimental meat	(2.10 ± 0.0) × 10^3^	(4.73 ± 0.26) × 10^3 a^	(4.30 ± 0.27) × 10^2 b^	(1.87 ± 0.06) × 10^5 c^	(4.10 ± 0.26) × 10^6^ ^d^ *
	15,000 CFU/mL	Control 1	(2.27 ± 0.06) × 10^3^	-	-	(1.97 ± 0.06) × 10^5^	(5.67 ± 0.29) × 10^6^
		Control 2	(2.17 ± 0.12) × 10^3^	(4.4± 0.61) × 10^3^	-	(2.0 ± 0.0) × 10^5^	(5.80 ± 0.53) × 10^6^
		Experimental meat	(2.2 ± 0.1) × 10^3^	(1.2 ± 0.1) × 10^4^ ^a^	(1.0 ± 0.17) × 10^3 b^	(1.87 ± 0.06) × 10^5 c^	(4.33 ± 0.21) × 10^6^ ^d^ *

Note: ^a–d^ superscripts denote statistically different values in rows and * denotes statistically different values in columns.

**Table 2:** The effect of anolyte’s concentration on the *L. monocytogenes* count of meat cuts contaminated with *L. monocytogenes*.

Concentration of Anolyte Used for Spraying of Meat Cuts	Suspension of *L. monocytogenes* Used for Contamination of Meat Cuts, CFU/mL	Object of Investigation (Raw Meat)	*L. monocytogenes*. CFU/g Meat Cut				
			Raw Meat	Meat after Contamination	Meat after Spraying with Anolyte	After 10 Days of Incubation at 0–4 °C	After 29 Days of Incubation at 0–4 °C
20%, beef	1500 CFU/mL	Control 1	<1.0 × 10^1^	-	-	<1.0 × 10^1^	<1.0 × 10^1^
		Experimental meat	<1.0 × 10^1^	(3.87 ± 0.15) × 10^3 a^	<1.0 × 10^1 b^	<1.0 × 10^1 b^	<1.0 × 10^1 b^
	15,000 CFU/mL	Control 1	<1.0 × 10^1^	-	-	<1.0 × 10^1^	<1.0 × 10^1^
		Control 2	<1.0 × 10^1^	(3.17 ± 0.35) × 10^3 a^	-	(2.97 ± 0.42) × 10^3 a^	(2.67 ± 0.49) × 10^3 b^
		Experimental meat	<1.0 × 10^1^	(8.77 ± 0.59) × 10^3 a^	(3.33 ± 0.58) × 10^2 b^	<1.0 × 10^1 c^	<1.0 × 10^1 c^

Note: ^a–c^ superscripts denote statistically different values in rows.

**Table 3:** The effect of anolyte’s concentration on the total viable count of meat cuts contaminated with *S.* Typhimurium.

Concentration of Anolyte Used for Spraying of Meat Cuts	Suspension of *S.* Typhimurium Used for Contamination of Meat Cuts, CFU/mL	Object of Investigation (Raw Meat)	Total Viable Count. CFU/g Meat Cut				
			Raw Meat	Meat after Contamination	Meat after Spraying with Anolyte	After 10 Days of Incubationat 0–4 °C	After 29 Days of Incubation at 0–4 °C
20%, beef	1500 CFU/mL	Control 1	(2.07± 0.06) × 10^3^	-	-	(2.63 ± 0.06) × 10^5^	(6.43 ± 0.25) × 10^6^
		Experimental meat	(2.10 ± 0.00) × 10^3^	(4.73 ± 0.06) × 10^3 a^	(5.83 ± 0.21) × 10^2 b^	(2.40 ± 0.1) × 10^5 c^ *	(4.03 ± 0.06) × 10^6^ *
	15,000 CFU/mL	Control 1	(2.07 ± 0.06) × 10^3^	-	-	(2.63 ± 0.06) × 10^5^	(6.43 ± 0.25) × 10^6^
		Control 2	(2.17 ± 0.06) × 10^3^	(4.4 ± 0.1) × 10^3 a^	-	(2.63 ± 0.06) × 10^5 b^	(6.6 ± 0.26) × 10^6 c^
		Experimental meat	(2.13 ± 0.15) × 10^3^	(1.1 ± 0.06) × 10^4 a^	(1.1 ± 0.06) × 10^3 b^	(2.43 ±0.06) × 10^5 c^	(4.7 ± 0.1) × 10^6^ ^d^ *

Note: ^a–d^ superscripts denote statistically different values in rows and * denotes statistically different values in columns.

**Table 4:** The effect of anolyte’s concentration on the *S.* Typhimurium count of meat cuts contaminated with *S.* Typhimurium.

Concentration of Anolyte Used for Spraying of Meat Cuts	Suspension of *S.* Typhimurium Used for Contamination of Meat Cuts, CFU/mL	Object of Investigation (Raw Meat)	Total Viable Count. CFU/g Meat Cut				
			Raw Meat	Meat after Contamination	Meat after Spraying with Anolyte	After 10 Days of Incubation at 0–4 °C	After 29 Days of Incubation at 0–4 °C
20%, beef	1500 CFU/mL	Control 1	<1.0 × 10^1^	-	-	<1.0 × 10^1^	<1.0 × 10^1^
		Experimental meat	<1.0 × 10^1^	(4.3 ± 0.3) × 10^3 a^	<1.0 × 10^1 b^	<1.0 × 10^1 b^	< 1.0 × 10^1 b^
	15,000 CFU/mL	Control 1	<1.0 × 10^1^	-	-	<1.0 × 10^1^	<1.0 × 10^1^
		Control 2	<1.0 × 10^1^	(3.4 ± 0.26) × 10^3 a^	-	(3.07 ± 0.31) × 10^3 a^	(2.83 ± 0.21) × 10^3 a^
		Experimental meat	<1.0 × 10^1^	(1.0 ± 0.8) × 10^4 a^	(4.3 ± 0.06) × 10^2 b^	<1.0 × 10^1 c^	<1.0 × 10^1 c^

Note: ^a–c^ superscripts denote statistically different values in rows.

**Table 5:** Effect of anolyte on the total viable count of minced pork contaminated by *L. monocytogenes*.

Contamination Level	Total Viable Count, CFU/g				
	Raw Minced Pork	Raw Pork Contaminated by *L. monocytogenes*	Contaminated Raw Pork after Addition of 18% Undiluted Anolyte and Mixing	Contaminated Pork Mixed with Anolyte after 10 Days of Incubation at 0–4 °C	Contaminated Pork Mixed with Anolyte after 29 Days of Incubation at 0–4 °C
10 mL 1.5 × 10^3^ CFU/mL suspension of *L. monocytogenes*	(2.20 ± 0.10) × 10^4^	(1.82 ± 0.10) × 10^4 a^	(8.02 ± 0.10) × 10^3 b^	(1.40 ± 0.10) × 10^4 a^	(2.80 ± 0.17) × 10^5 c^
10 mL 1.5 × 10^4^ CFU/mL suspension of *L. monocytogenes*	(2.20 ± 0.10) × 10^4^	(2.40 ± 0.20) × 10^4 a^	(1.40 ± 0.17) × 10^4 b^ *	(1.80 ± 0.20) × 10^4 b^	(4.10 ± 0.10) × 10^5 c^ *
10 mL 1.5 × 10^5^ CFU/mL suspension of *L. monocytogenes*	(0.70 ± 0.10) × 10^4^	(6.60 ± 0.26) × 10^4 a^	(2.70 ± 0.20) × 10^4 b^ **	(1.80 ± 0.10) × 10^4 c^	(8.00 ± 0.20) × 10^5 d^ **

Note: ^a–d^ superscripts denote statistically different values in rows and * and ** denote statistically different values in columns.

**Table 6:** Effect of anolyte on the *L. monocytogenes* count of minced pork contaminated by *L. monocytogenes*.

Contamination Level	*L. monocytogenes* Count. CFU/g				
	Raw Minced Pork	Raw Pork Contaminated by *L. monocytogenes*	Contaminated Raw Pork after Addition of 18% Undiluted Anolyte and Mixing	Contaminated Pork Mixed with Anolyte after 10 Days of Incubation at 0–4 °C	Contaminated Pork Mixed with Anolyte after 29 Days of Incubation at 0–4 °C
10 mL 1.5 × 10^3^ CFU/mL suspension of *L. monocytogenes*	<1.00 × 10^1^	(1.60 ± 0.10) × 10^3 a^	<1.00 × 10^1 b^	<1.00 × 10^1 b^	<1.00 × 10^1 b^
10 mL 1.5 × 10^4^ CFU/mL suspension of *L. monocytogenes*	<1.00 × 10^1^	(3.70 ± 0.17) × 10^3 a^	(6.00 ± 0.10) × 10^2 b^	(5.60 ± 0.17) × 10^2 b^	<1.00 × 10^1 c^
10 mL 1.5 × 10^5^ CFU/mL suspension of *L. monocytogenes*	<1.00 × 10^1^	(2.50 ± 0.10) × 10^4 a^	(1.30 ± 0.20) × 10^3 b^	(1.80 ± 0.26) × 10^3 b^	(2.20 ± 0.20) × 10^2 c^ *

Note: ^a–c^ superscripts denote statistically different values in rows and * denotes statistically different values in columns.

**Table 7:** Effect of anolyte on the total viable count of minced pork contaminated by *S.* Typhimurium.

Contamination Level	Total Viable Count, CFU/g				
	Raw Minced Pork	Raw Pork Contaminated by *S.* Typhimurium	Contaminated Raw Pork after Addition of 18% Undiluted Anolyte and Mixing	Contaminated Pork Mixed with Anolyte after 10 Days of Incubation at 0–4 °C	Contaminated Pork Mixed with Anolyte after 29 Days of Incubation at 0–4 °C
10 mL 1.5 × 10^3^ CFU/mL suspension of *S.* Typhimurium	(2.20 ± 0.10) × 10^4^	(2.20 ± 0.26) × 10^4 a^	(6.40 ± 0.17) × 10^3 b^	(2.10 ± 0.17) × 10^4 a^	(4.10 ± 0.20) × 10^5 c^
10 mL 1.5 × 10^4^ CFU/mL suspension of *S.* Typhimurium	(2.20 ± 0.10) × 10^4^	(5.40 ± 0.26) × 10^4 a^	(1.90 ± 0.10) × 10^4 b^	(2.30 ± 0.26) × 10^4 b^	(4.00 ± 0.17) × 10^5 c^
10 mL 1.5 × 10^5^ CFU/mL suspension of *S.* Typhimurium	(0.70 ± 0.11) × 10^4^	(5.40 ± 0.17) × 10^4 a^	(3.60 ± 0.36) × 10^4 b^	(3.90 ± 0.17) × 10^4 b^	(7.80 ± 0.26) × 10^5 c^ *

Note: ^a–c^ superscripts denote statistically different values in rows and * denotes statistically different values in columns.

**Table 8:** Effect of anolyte on the *S*. Typhimurium count of minced pork contaminated by *S.* Typhimurium.

Contamination Level	*S.* Typhimurium. CFU/g				
	Raw Minced Pork	Raw Pork Contaminated by *S.* Typhimurium	Contaminated Raw Pork after Addition of 18% Undiluted Anolyte and Mixing	Contaminated Pork Mixed with Anolyte after 10 Days of Incubation at 0–4 °C	Contaminated Pork Mixed with Anolyte after 29 Days of Incubation at 0–4 °C
10 mL 1.5 × 10^3^ CFU/mL suspension of *S.* Typhimurium	<1.00 × 10^1^	(3.50 ± 0.43) × 10^2 a^	<1.00 × 10^1 b^	<1.00 × 10^1 b^	<1.00 × 10^1 b^
10 mL 1.5 × 10^4^ CFU/mL suspension of *S.* Typhimurium	<1.00 × 10^1^	(3.00 ± 0.17) × 10^3 a^	(1.30 ± 0.20) × 10^3 b^	(1.90 ± 0.20) × 10^3 b^	(8.0 ± 0.08) × 10^1 c^ *
10 mL 1.5 × 10^5^ CFU/mL suspension of *S.* Typhimurium	<1.00 × 10^1^	(1.90 ± 0.17) × 10^4 a^	(1.40 ± 0.17) × 10^3 b^	(2.00 ± 0.26) × 10^3 b^	(2.30 ± 0.20) × 10^2 c^ **

Note: ^a–c^ superscripts denote statistically different values in rows and * and ** denote statistically different values in columns.

## Data Availability

The data that support the findings of this study are available from the corresponding author, R.R., upon reasonable request.
